# The Political Glass Cliff: When Ethnic, Racial and Immigration Minority Participants Choose Minority Candidates for Hard‐To‐Win Seats

**DOI:** 10.1002/casp.70014

**Published:** 2024-11-20

**Authors:** Cristina Aelenei, Yvette Assilaméhou‐Kunz, Vincenzo Iacoviello, Clara Kulich

**Affiliations:** ^1^ Laboratoire de Psychologie Sociale Université Paris Cité Boulogne‐Billancourt France; ^2^ Institut de Recherche Médias, Cultures, Communication et Numérique, University of Sorbonne Nouvelle Paris France; ^3^ Faculty of Psychology and Educational Sciences University of Geneva Geneva Switzerland

**Keywords:** change potential, ethnic minority, glass cliff, intra‐minority solidarity, politics

## Abstract

Atypical political candidates, such as those from ethnic, racial and immigration (ERI) minorities (vs. majority), are more likely to be chosen for hard‐to‐win seats than easy‐to‐win seats, a phenomenon known as the political glass cliff. This research aimed to uncover how the ERI status of decision makers played a role in this process. We hypothesised the emergence of a glass cliff pattern, that is, the preference for an ERI minority candidate over an ERI majority candidate for a hard‐to‐win seat, particularly among ERI minority participants, which are likely to perceive greater electoral potential in the ERI minority candidate compared to majority participants. Across two scenario‐based experiments (Study 1: *N* = 264; Study 2: *N* = 375), ERI minority and majority participants played the role of political party leaders and made decisions regarding candidate nominations either in easy‐to‐win or in hard‐to‐win electoral districts. In Study 1, ERI minority participants, but not ERI majority participants, were more likely to choose an ERI minority (vs. majority) candidate for hard‐to‐win seats. Moreover, ERI minority participants made stronger attributions of change potential, competence and communion to ERI minority (vs. majority) candidates, suggesting that intra‐minority solidarity could play a role in their choice. Although this result did not replicate in Study 2, exploratory analyses revealed a consistent glass cliff pattern among ERI minority men in both studies. Please refer to the Supplementary Material section to find this article's Community and Social Impact Statement.

Ethnic, racial and immigrant (ERI) minorities remain underrepresented in politics in many European nations (Morales and Vincent‐Mory 2022). Because data collection and monitoring of ethnicity is very restricted in Europe (with the exception of the United Kingdom, Finland and Norway; Directorate‐General for Justice and Consumers and Huddleston 2017), statistics on ERI status are difficult to gather and are often based on proxies, using, for example, candidate names (Directorate‐General for Justice and Consumers and Farkas 2017). Estimates suggest that at least 10% of the European population are racial and ethnic minorities but only 5% of the members of European parliament belong to these groups (European Network Against Racism 2019). In France, where the present research took place, although statistics on ERI minorities are not officially recorded, an estimated 11% of the population are members of ERI minorities, more than double the percentage in the political sphere (Brutel 2017). In 2002 the National Assembly had no ERI minority representatives (Bird 2003). Between 2012 and 2017, the ERI proportion tripled and then stagnated at 5.8% in 2022 (Houeix and Makooi 2022). These estimates suggest that, despite ERI minorities still being underrepresented in politics, their representation has tended to rise over time. Thus, the circumstances, which prevent or facilitate changes in the representation of minorities, need to be studied.

Several scholars of political sciences have proposed that ERI minority underrepresentation may be due to sociopolitical dynamics involving both voters' biases and political parties' choices (Auer, Portmann, and Tichelbaecker 2023), with some scholars arguing that the role of the party is more decisive (Keslassy 2009). Thus, if we want to get a better understanding of when and why ERI minority candidates appear on the political landscape, we need to focus on when and why they are chosen as political candidates for their party. In the present research, we will focus on one particular phenomenon: the over‐proportional selection of underrepresented groups to run in hard‐to‐win electoral seats, a phenomenon known as a political glass cliff (Ryan, Haslam, and Kulich 2010). Although glass cliff nominations for ERI minority groups have been illustrated with observational data from elections in the United Kingdom and France (Kulich, Ryan, and Haslam 2014; Robinson et al. 2024), little is known about the motives that underpin these choices. One exception is experimental research that revealed that decision makers with left‐wing political orientation likely make glass cliff choices of ERI minority candidates (Aelenei et al. 2020). These results deliver initial social psychological information on potentially benign motives that value ERI minority nominations, particularly in hard‐to‐win seats.

By employing an experimental methodology, our research aimed to gain further insights into the characteristics of individuals who are more likely to nominate ERI minority (vs. majority) candidates and the reasons behind their choices. We used scenarios involving electoral seats that are either easier or harder to win to test whether the decision maker's own ERI (minority or majority) affiliation could predict their choices in these scenarios. Specifically, we hypothesised that individuals identifying as an ERI minority might, similarly to left‐wing voters (Aelenei et al. 2020), value ERI minority candidates in hard‐to‐win contexts. The presence of the glass‐cliff pattern in leadership appointments has been demonstrated across both expert and general populations (e.g., Haslam and Ryan 2008; Morgenroth et al. 2020), which highlights that some basic social psychological dynamics seem to be at play. We thus invited non‐expert participants to enact the role of political party representatives and to make candidate choices.

## 1 The Political Glass Cliff

Our particular attention to seat winnability (easy‐ or hard‐to‐win) in ERI minority candidate nominations derives from research on the glass cliff phenomenon that illustrates that women or ERI minority individuals are more likely to rise to positions of power in precarious contexts compared to men or ERI majorities (Kulich, Ryan, and Haslam 2014; Ryan et al. 2016). According to a meta‐analysis, this phenomenon is particularly prominent in the political context where minority individuals are more likely to run for hard‐to‐win seats, that is, seats where their political party has not succeeded in the past and thus is less likely to win them in the future (Morgenroth et al. 2020). Illustrations range from archival studies on national elections in different countries to experimental evidence showing a political glass cliff for women and ERI minority groups (Kulich, Ryan, and Haslam 2014; Robinson et al. 2021; Ryan, Haslam, and Kulich 2010; Thomas and Bodet 2013).

The motivations for glass cliff appointments may vary and are context‐specific as regards decision makers' characteristics (e.g., their endorsement of stereotypes and prejudice and their political orientation), the institutional context (e.g., politics and business) and the group concerned (e.g., women and ERI minority individuals). Some proposed that, in difficult times, minority candidates may serve as scapegoats (Acar and Sümer 2018; Ryan and Haslam 2005) to protect majority candidates from failure or to meet social demands for more diversity (Kulich, Ryan, and Haslam 2014; Robinson et al. 2021). Although these hostile motivations may explain some glass cliff situations, evidence is sparse and it appears that in many crisis situations, decision makers seek solutions to overcome the adverse circumstances. Research in the business context shows that struggling organisations are thus often looking for a change, and the atypicality of minority candidates may reflect an unconventional or different type of leadership (Reinwald, Zaia, and Kunze 2023). Thus, signalling to stakeholders that change is on the way, or choosing a leader who is believed to have a different take on the task than traditional (White male) leaders, can also be a valid motivation behind the choice of a minority individual (Bruckmüller and Branscombe 2010; Kulich et al. 2015; Ryan et al. 2016). Such ‘benign’ reasoning is likely to be followed by certain types of decision makers. Experimental research has indeed demonstrated that individuals with a left‐wing political orientation (e.g., individuals with more egalitarian worldviews and more positive attitudes toward minority groups) were more likely than right‐wing individuals to select an ERI minority candidate (vs. majority candidate) to compete for hard‐to‐win seats (vs. easy‐to‐win seats; Aelenei et al. 2020). This research further revealed that left‐wing oriented individuals were more likely than their right‐wing counterparts to associate an ERI minority candidate (vs. a majority candidate) with a potential to signal and implement change. Thus, when decision makers from a left‐wing party need to turn the situation around (i.e., because of their negative history in past elections), they are more likely to select a candidate who is associated with a greater potential for change. In sum, there are indications that political glass cliff appointments can be motivated by a genuine belief that ERI minority candidates could be a better solution for turning around a district where the party traditionally struggles, compared to traditional ERI majority candidates.

## 2 ERI Minority Members as Political Decision Makers

Beyond political orientation, a characteristic that may be influencing the choice of ERI minority candidates is the decision makers' own ERI background. Existing evidence suggests that when ERI minority members are political decision makers, they not only favour members of their specific ERI subgroup (i.e., ingroup favouritism), but ERI minorities in general. Based on Canadian archival data, Tolley (2019) showed that in districts with a racialized (vs. White) local party president, more racialized minority candidates were nominated in the list of candidates running for election. Van Trappen, Vandeleene, and Wauters (2021) found a similar ‘spillover’ effect studying the role of immigrant (vs. non‐immigrant) origins of heads of running lists who acted as selector of other candidates. Their analysis based on data from the 2018 local elections in Flanders (Belgium) showed that lists with an immigrant‐origin head of list had a higher presence of immigrant‐origin candidates.

In both studies, the minority group (i.e., a group with racialized or immigrant origins) was operationalised as a group of people with various ethnic, racial or immigrant backgrounds.^1^ An explanation for such a preference may be *intra‐minority solidarity* (sometimes also labelled *inter*‐minority solidarity or ‘rainbow coalition’; Besco 2015), which refers to attitudes and actions of a prosocial and coalitional nature from members of a minority group towards members of another minority group (e.g., Afro‐Americans towards Latin‐Americans, for a review see Burson and Godfrey 2020). Based on the common ingroup identity model (Gaertner et al. 1993), several studies have shown that minority group members show intra‐minority solidarity when they perceive a common identity with other minority groups based on their minority status within the society and/or similar discrimination or stigmatisation experiences (e.g., Craig and Richeson 2012, 2016; Glasford and Calcagno 2012; Macías Mejía 2023). According to this perspective, intra‐minority solidarity can be understood as an instance of extended ingroup solidarity, which can take many forms, such as support for policies to benefit the other minority group, willingness to engage in political action on behalf of the other minority group or positive attitudes towards the other minority group. For ERI minority political decision makers, intra‐minority solidarity can therefore be displayed through a more positive perception of an ERI minority candidate compared to a majority candidate, because the ERI minority candidate is more likely to be perceived as a highly suitable candidate, compared to a majority candidate.

We claim that this perceived advantage should be particularly relevant when the political situation of the party is precarious and past, more traditional, candidates have not been able to reverse the trend. Indeed, research suggests that crises elicit negative affect and the urge of fixing the situation (Brown, Diekman, and Schneider 2011). In this context, ERI minority decision makers might be more likely, compared to majority decision makers, to choose an ERI minority candidate, believing they have a better potential to turn the situation around. Such an atypical choice would be less relevant when the likelihood of winning the seat is high (i.e., when the past electoral history is favourable to the party). Thus, we hypothesise that ERI minority decision makers should be more likely than ERI majority decision makers to choose ERI minority candidates, especially when the seat is hard‐to‐win (vs. easy‐to‐win). In other words, we would expect to observe a glass cliff pattern among ERI minority decision makers, resulting from benevolent motivation and a positive perception of an ERI minority candidate.

## 3 The Present Research

In two experimental studies (an additional exploratory pilot study is reported in the Supporting Information S1: SM2, ^2^), we examined the role of decision maker ERI status on their glass cliff decisions regarding an ERI minority candidate nomination to run for a seat in the French National Assembly. Although candidates do not need to be affiliated with a party, most of them are. Parties differ in how they designate candidates. In most parties, an internal committee designates the candidates. These selections can either be final or require validation by a vote from party members. In other parties, local assemblies suggest candidates within their constituencies, and these suggestions are then validated by an internal committee.

In the French context, as in many other Western European countries, ethnic, racial or immigrant minority backgrounds are often conflated. ERI minorities are most of the time racial minorities and have an immigration background. The main ethnic minority groups (people with North African, sub‐Saharan African or Asian origins) are non‐White people with an immigrant background, as they are immigrants or descendants of immigrants. We investigated glass cliff decisions towards an ERI minority candidate with North African origins, as that is the largest minority group in the French context and one of the most discriminated against in various domains (Lê et al. 2022). Consistent with previous work on ERI minority political decision makers (Tolley 2019; Van Trappen, Vandeleene, and Wauters 2021), we considered decision‐maker ERI status regardless of specific minority subgroups. In addition, we postulated that ERI minority members in France would display intra‐minority solidarity towards an ERI minority candidate with North African origins, regardless of the specific ERI minority group to which they belonged themselves. Indeed, several surveys conducted in the French context showed that many minority group members shared the feeling of being discriminated against because of their skin colour or their immigrant origins (e.g., Brinbaum and Guégnard 2012). We formulated the following hypotheses concerning glass cliff decisions on the one hand, and the perception of a candidate on the other:

Hypothesis 1 *We predict a glass cliff in the sense that an ERI minority candidate will be more likely to be chosen in a hard‐to‐win district (compared to an easy‐to‐win district), specifically among ERI minority participants.*

Furthermore, to tap into the sociopsychological motivations for such choices, we hypothesised a mediated moderation model, as described below.

Hypothesis 2 *ERI minority participants will perceive the minority candidate more positively and as having more change potential compared to the majority candidate. This relative evaluation favouring the minority candidate will increase the probability of choosing the minority candidate over the majority candidate, particularly in a hard‐to‐win electoral district where these characteristics would be highly relevant (and arguably more relevant than in the easy‐to‐win district). We will explore similar mediated moderation effects as described in* H2 *with candidate traits (related to instrumental and relational competencies) as mediators.*

## 4 Study 1

### 4.1 Method

#### 4.1.1 Participants

A total of 313 students from an Institute of Technology (IUT) in Paris participated in the study. To achieve a larger sample size, data were collected over the course of 2 years: 2018 (*N* = 148) and 2019 (*N* = 165). The sample consisted of two consecutive cohorts of students enrolled in a psychology class. We excluded 49 participants: those under 18 years of age, those who did not give consent for their responses to be used for research and those who answered incorrectly to at least one of the two comprehension checks (explained below). The final sample consisted of 264 participants (167 women, 97 men; *M*_age_ = 18.52, SD_age_ = 0.68). For political orientation, measured on a scale from 1 (*left‐wing*) to 9 (*right‐wing*), our sample showed a normal distribution (*M* = 5.05, SD = 1.56).

We assessed ERI status through participants' perception of their own ethnic and immigration background. We first asked participants whether, concerning their ethnic background, they considered themselves to be: White, Black, North African (Maghrebi), Asian, Middle Eastern or Other (please specify). A subsequent question asked them whether they considered their ethnic group to be an ethnic minority in France (Yes/No). Finally, an item asked participants whether they considered themselves to have an immigrant origin (Yes/No). We assigned participants to the ERI minority group (*N* = 129) when they considered themselves as belonging to an ethnic minority group in France, or as having an immigrant background. Participants answering negatively to both questions were assigned to the ERI majority group (*N* = 132, 3 excluded due to missing data). In other words, according to the definition, our ERI minority category included participants who self‐identified as a disadvantaged ethnic group (minority) or as having an immigrant background. We did not include participants who only indicated belonging to a non‐White ethnic group while responding in the negative to the ERI minority self‐identification and immigration items because they likely did not perceive themselves as being part of ERI minority groups. According to our theoretical stance, intra‐ethnic solidarity would require that participants self‐identify as having a minority status and thus as being part of the larger ERI minority community.

#### 4.1.2 Procedure

The study used a paper‐pencil questionnaire, which was administered at the end of a regular lecture. We adopted the scenario‐based procedure outlined by Aelenei et al. (2020). Students were provided with general instructions, asking them to imagine themselves in the role of Party X's leader. In this capacity, their task was to nominate a candidate in a specified electoral district (i.e., District A) for the upcoming legislative elections. Depending on the experimental condition, the district represented either an ‘easy‐to‐win’ (*N* = 128) or a ‘hard‐to‐win’ (*N* = 136) seat for Party X. In the ‘easy‐to‐win’ condition, a graph was presented to participants, indicating that Party X had won the last elections in the district with 54.6% of the vote, surpassing Party Y, which garnered 45.4%. Conversely, in the ‘hard‐to‐win’ condition, the graph showed that Party X had lost the previous election, securing only 45.4% of the vote, with Party Y winning at 54.6% (an English translation of the scenarios can be found in the Supporting Information S1: SM3). The first comprehension check item assessed whether the participants correctly read the graph and understood which party (X or Y) had won the last election in the district. The second comprehension check investigated participants' assessment regarding the chances for Party X to win the next elections in the district. Specifically, the item asked which party had the highest chances of winning in the district: Party X, Party Y, both parties or neither party. For participants in the ‘easy‐to‐win’ condition (i.e., district where Party X won the previous elections), incorrect answers were ‘Party Y’ or ‘neither party had the highest chances to win’. In the ‘hard‐to‐win’ condition (i.e., district where Party X lost the previous elections), incorrect answers were ‘Party X’ or ‘neither party had the highest chances to win’.

Next, participants were informed that they would receive a short description of two candidates. As the leader of Party X, their responsibility would be to evaluate both candidates and then nominate one of them to run as Party X's representative for an electoral seat in District A. To enhance participants' engagement with the task, they were also told: ‘We understand that judging someone based solely on the information we are about to present can be challenging. However, please note that we are specifically interested in your spontaneous impressions.’ Participants then read the descriptions of the two candidates, inspired by Aelenei et al. (2020). These descriptions were crafted to be equivalent in terms of education (i.e., a master's degree in political science), professional career (i.e., management‐level employment) and hobbies. The distinguishing feature between the two candidates was their ERI status. The ERI minority candidate had a typically North African first and last name (Abdel Benzekri), was born in Algeria, but had resided in France since he was five—highlighting his upbringing and education in France. In contrast, the ERI majority candidate, with a distinctive French first and last name (Olivier Fournier), was born in France and had lived there his entire life. The candidates' descriptions were presented in a counterbalanced order. Following this, participants were prompted to evaluate each candidate on various measures and select the candidate they felt was best suited for District A. They then completed a series of sociodemographic questions covering gender, age, nationality, ethnic background, immigrant background, political orientation and their interest and engagement in French politics. The questionnaire concluded with a comprehensive restatement of ethical consent. An overview of additional measures that are not relevant for the present paper can be found in the Supporting Information S1: SM4.

#### 4.1.3 Measures

Participants responded to the measures on 7‐point Likert scales.

Candidate Traits. To assess positive candidate views, participants evaluated each candidate on nine traits that referred to the candidates' competence, sociability and morality (Leach, Ellemers, and Barreto 2007), which are the main dimensions of person impression. To ensure that the trait dimensions were understood in the same manner for both candidates, we conducted two separate exploratory factor analyses (extraction method: principal axis factoring, with a direct oblimin rotation). For each candidate, we identified a two‐factor pattern: the first factor focused on competence (competent, intelligent and skilled; *α*_Olivier_ = 0.84, *α*_Abdel_ = 0.86; *M*_Olivier_ = 5.54, SD_Olivier_ = 0.89, *M*_Abdel_ = 5.60, SD_Abdel_ = 0.97). The second factor, relating to communion, grouped sociability and morality traits together, including likable, friendly, warm, honest, sincere and trustworthy (*α*_Olivier_ = 0.91, *α*_Abdel_ = 0.91; *M*_Olivier_ = 4.66, SD_Olivier_ = 1.06, *M*_Abdel_ = 5.01, SD_Abdel_ = 1.02).

Change Potential. To assess the change potential associated with each candidate, we included seven items (adapted from Aelenei et al. 2020) that captured both the actual change that the candidate could implement and the symbolic change that the candidate could signal. For each candidate, we conducted an exploratory factor analysis with a direct oblimin rotation. We eliminated items with communalities and factor loadings below 0.40 (Costello and Osborne 2005). Consequently, Items 1, 2 and 7 were excluded from both analyses. The four remaining items, namely the candidate ‘will signal to voters that Party X wants things to change’, ‘will show to the public and other parties that Party X has adopted changes in the way the party is run’, ‘will conduct his political campaign in this district differently from his predecessors’, and ‘is likely to introduce new and different ideas compared to his predecessors’ loaded on a single factor in the assessments of both candidates. This suggests that these items capture a general change potential associated with each candidate (*α*_Olivier_ = 0.73, *α*_Abdel_ = 0.77; *M*_Olivier_ = 4.43, SD_Olivier_ = 1.04, *M*_Abdel_ = 5.01, SD_Abdel_ = 1.03).

Choice of the Candidate. The participants were asked to indicate whom they would choose as the nominee of their party to run for election in District A (minority vs. majority candidate).

### 4.2 Results for Main Hypotheses Tests

#### 4.2.1 Choice of Candidate

The choice of candidate as the dependent variable was coded as: 1 = choice of minority candidate Abdel and 0 = choice of majority candidate Olivier.^3^ As main predictors, we considered type of electoral seat (−0.5 = hard‐to‐win; 0.5 = easy‐to‐win), participants' ERI status (−0.5 minority; 0.5 = majority) and the interaction term.

**FIGURE 1:**
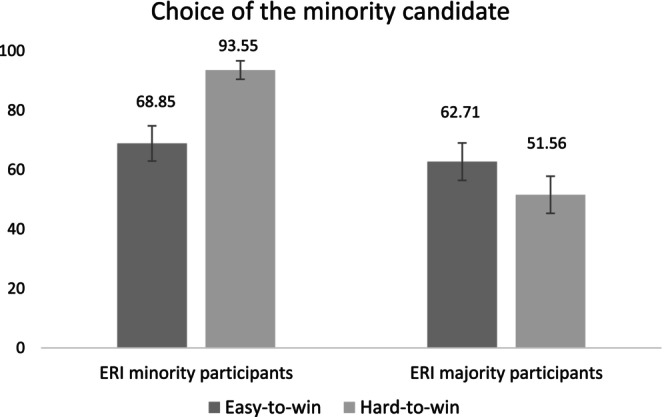
Estimated percentages with standard errors for the choice of the ERI minority candidate by type of seat and participant ERI status in Study 1.

The results showed two main effects and two interaction effects. The main effect of type of seat, *B* = −0.71, 95% CI [−1.44, −0.06], *χ* ^2^(1, *n* = 246) = 4.23, *p* = 0.040, odds ratio (OR) = 0.49, showed that participants were more likely to choose the minority candidate in the hard‐to‐win electoral district (79.71%) than in the easy‐to‐win electoral district (65.85%). The main effect of participants' ERI status, *B* = −1.44, 95% CI [−2.17, −0.80], *χ* ^2^(1, *n* = 246) = 17.39, *p* < 0.001, OR = 0.24, showed that minority participants (84.99%) were more likely compared to majority participants (57.23%) to choose the minority candidate. As predicted in H1, the interaction effect between ERI status and type of seat was significant, *B* = 2.34, 95% CI [1.05, 3.80], *χ* ^2^(1, *n* = 246) = 11.42, *p* = 0.001, OR = 10.36 (Figure 1). Simple effects analyses showed that minority participants were more likely to choose the minority candidate in the hard‐to‐win (versus the easy‐to‐win) electoral district, *B* = −1.88, 95% CI [−3.17, −0.82], *χ* ^2^(1, *n* = 246) = 10.29, *p* = 0.001, OR = 0.15. The effect of type of seat was not significant among majority participants, *B* = 0.45, 95% CI [−0.26, 1.19], *χ* ^2^(1, *n* = 246) = 1.55, *p* = 0.213, OR = 1.58. Moreover, minority participants were more likely, compared to majority candidates, to choose the minority candidate for the hard‐to‐win electoral district, *B* = −2.61, 95% CI [−3.89, −1.58], *χ* ^2^(1, *n* = 246) = 20.68, *p* < 0.001, OR = 0.07. No effect of the ERI status was found for the easy‐to‐win electoral district, *B* = −0.27, 95% CI [−1.04, 0.48], *χ* ^2^(1, *n* = 246) = 0.50, *p* = 0.479, OR = 0.76.^4^

#### 4.2.2 Candidate Evaluations

For the subsequent dependent variables, we conducted linear regression analyses, with type of seat, participant ERI status and the interaction term as predictors. To obtain the relative evaluation of the ERI minority candidate compared to the majority candidate, we created difference scores (i.e., change potential or traits associated with the minority candidate—change potential or traits associated with the majority candidate).

**FIGURE 2:**
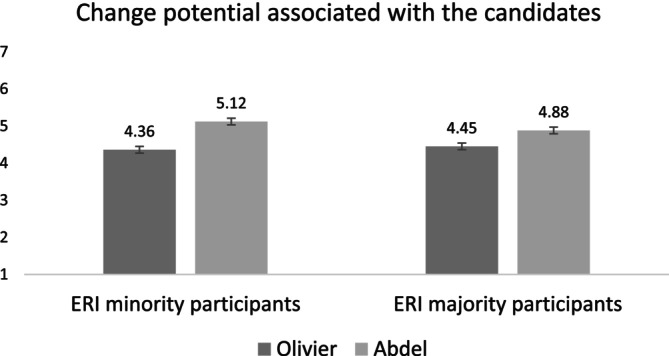
Means and standard errors for the change potential associated with the candidates as a function of participant ERI status in Study 1.

Change Potential. Regressing the difference score on the predictors revealed that overall, the participants evaluated Abdel (i.e., the minority candidate) as having a higher change potential compared to Olivier (i.e., the majority candidate), *B* = 0.59, 95% CI [0.44, 0.74], *t*(256) = 7.83, *p* < 0.001, *η* ^2^_*p*_ = 0.19. This difference in favour of Abdel was stronger among minority participants (*M* = 0.75, *SE* = 0.11, *p* < 0.001) than majority participants (*M* = 0.43, *SE* = 0.11, *p* < 0.001), *B* = −0.32, 95% CI [−0.62, −0.02], *t*(256) = −2.13, *p* = 0.034, *η* ^2^_*p*_ = 0.02. The means and standard errors are displayed in Figure 2.

**FIGURE 3:**
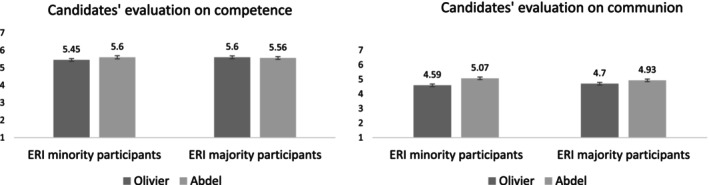
Means and standard errors for the competence and the communion evaluation of candidates as a function of participant ERI status in Study 1.

Candidate Trait. Analysis showed a similar results pattern on competence and on communion. We found an effect of participant ERI status on candidates' relative evaluation, competence: *B* = −0.20, 95% CI [−0.38, −0.03], *t*(256) = −2.36, *p* = 0.019, *η*_*p*_^2^ = 0.02; communion: *B* = −0.26, 95% CI [−0.47, −0.04], *t*(256) = −2.35, *p* = 0.019, *η* ^2^_*p*_ = 0.02, showing that compared to majority participants, minority participants evaluated Abdel as more competent and more communal than Olivier (Figure 3). We also found an effect of type of seat, competence: *B* = 0.24, 95% CI [0.07, 0.41], *t*(256) = 2.72, *p* = 0.007, *η* ^2^_*p*_ = 0.03; communion: *B* = 0.40, 95% CI [0.19, 0.62], *t*(256) = 3.73, *p* < 0.001, *η* ^2^_*p*_ = 0.05, showing that, compared to the participants in the hard‐to‐win condition, those in the easy‐to‐win condition perceived Abdel as more competent and more communal than Olivier.

#### 4.2.3 Mediation Analyses

**FIGURE 4:**
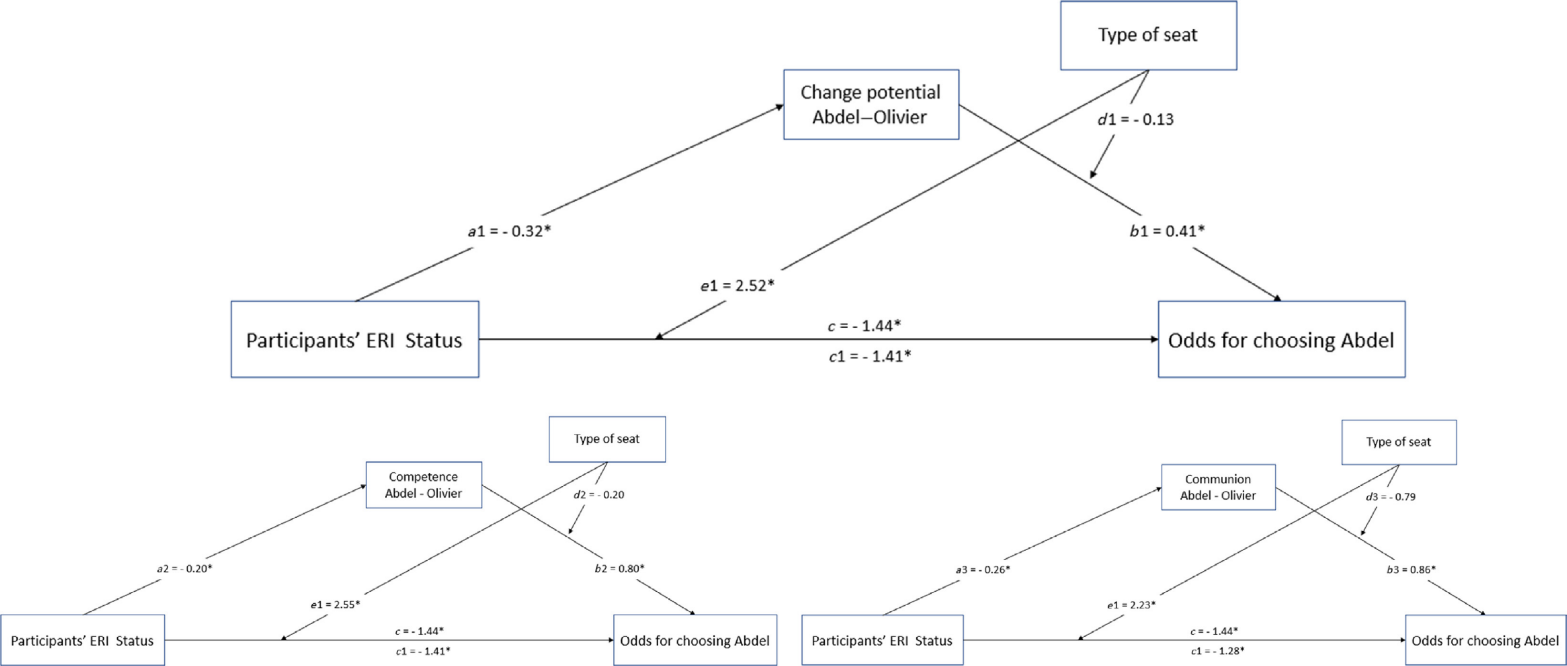
The moderated mediation models with change potential and traits as mediators. *Note:* Participant ERI status was coded: Minority = −0.5; majority = 0.5. Type of seat was coded: Hard‐to‐win = −0.5; easy‐to‐win = 0.5. The unstandardized paths are reported. ‘*’ flags significance. The *c* path represents the total effect of participant ERI status on choice. The *c*1 path represents the direct effect of participant ERI status on choice, controlling for the mediator.

To explain the glass‐cliff pattern observed among minority participants (i.e., higher odds to choose the minority candidate, Abdel, in the hard‐to‐win condition than in the easy‐to‐win condition), we considered the relative evaluation of the candidates on change potential (i.e., the difference score) as mediator, thereby testing H2. We reasoned that the minority participants were more likely than the majority participants to choose Abdel over Olivier because they perceived Abdel as having more change potential than Olivier. This should particularly occur in the hard‐to‐win condition where these characteristics would be highly relevant (and arguably more relevant than in the easy‐to‐win condition). Two further mediation models with similar reasoning considered competence and communion as mediators. In sum, we conducted three mediated moderation analyses, adopting the component approach based on joint‐significance tests (Yzerbyt et al. 2018). The models tested are depicted in Figure 4. This approach implies that mediated moderation is demonstrated if two tests are simultaneously significant: (1) the independent variable (i.e., ERI status) has a significant effect on the mediator (i.e., change potential, competence and communion; Paths *a*1, *a*2 and *a*3), and (2) the moderator (i.e., type of seat) significantly moderates the effect of the mediator on the outcome (i.e., choice of the ERI minority candidate, Paths *d*1, *d*2 and *d*3). We have already documented that ERI status had significant effects on change potential (*a*1 path), competence (*a*2 path) and communion (*a*3 path), namely that minority participants (more so than the majority participants) perceived Abdel as having more change potential, and as being more competent and more communal, than Olivier. Thus, we proceeded with testing the second part of the model (i.e., *d*1, *d*2 and *d*3 paths). The results did not support a mediated moderation model. The type of seat did not moderate the effects of change potential, competence or communion on the choice of the minority candidate (*p*s > 0.131). Instead, simple partial mediations were supported, suggesting that irrespective of type of seat, minority participants were more likely (compared to majority participants) to choose Abdel over Olivier because they perceived Abdel as having greater change potential and as being more competent and more communal than Olivier (*p*_*b*1_ = 0.013, *p*_*b*2_ < 0.001, *p*_*b*3_ *<* 0.001).

### 4.3 Exploratory Analyses: Participant's Gender and Political Orientation

**FIGURE 5:**
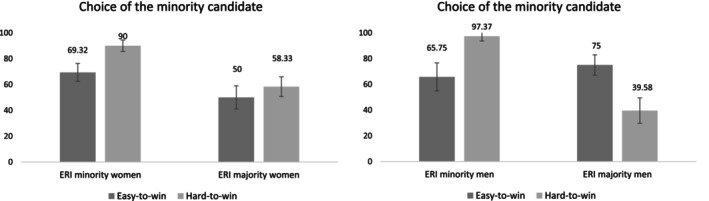
Estimated percentages with standard errors for the choice of the ERI minority candidate by type of seat and participant ERI status among women and men in Study 1.

As both candidates were men, we reasoned that participants' gender could play a role in their evaluation. Thus, in exploratory analyses that have not been preregistered, we also added participants' gender (coded: −0.5 = woman; 0.5 = man) and all of the interaction terms as predictors in our models. For the dependent variable choice of candidate, due to the small sample sizes across cells (some even null), we conducted an exact logistic regression, using the “logistf” package in R (the results are presented in Table 1). This analysis revealed that the interaction effect between ERI status and type of seat was moderated by participant gender, *B* = 3.43, 95% CI [0.38, 8.57], *χ* ^2^(1, *n* = 246) = 4.95, *p* = 0.026, OR = 30.96. The estimated percentages for the choice of the minority candidate, based on type of seat and ERI status, are presented separately for women and men participants in Figure 5. Simple effects analyses showed that the interaction effect between ERI status and type of seat was significant among male participants, *B* = 4.48, 95% CI [1.88, 9.47], *χ* ^2^(1, *n* = 246) = 13.67, *p* < 0.001, OR = 88.01. Specifically, minority male participants were more likely to choose the minority candidate in the hard‐to‐win (vs. the easy‐to‐win) electoral district, *B* = −2.96, 95% CI [−7.86, −0.68], *χ* ^2^(1, *n* = 246) = 7.27, *p* = 0.007, OR = 0.05, whereas majority male participants were more likely to choose the minority candidate in the easy‐to‐win (versus the hard‐to‐win) electoral district, *B* = 1.52, 95% CI [0.39, 2.73], *χ* ^2^(1, *n* = 246) = 7.03, *p* = 0.008, OR = 4.58. For female participants, the interaction effect between ERI status and type of seat did not reach significance, *B* = 1.05, 95% CI [−0.41, 2.60], *χ* ^2^(1, *n* = 246) = 1.95, *p* = 0.162, OR = 2.85. A main effect of type of seat showed that women were more likely to choose the minority candidate in the hard‐to‐win (vs. easy‐to‐win) electoral district, *B* = −0.86, 95% CI [−1.64, −0.13], *χ* ^2^(1, *n* = 246) = 5.40, *p* = 0.02, OR = 0.42. Furthermore, a main effect of ERI status showed that minority women were more likely to choose the minority candidate compared to majority women, *B* = −1.34, 95% CI [−2.12, −0.61], *χ* ^*2*^(1, *n* = 246) = 13.59, *p* < 0.001, OR = 0.26.

**TABLE 1:** Logistic regression results for type of electoral seat, participants' ERI status, and gender on candidate choice in Study 1.

	*B*	SE (*B*)	95% CI	*p*
TypeElectoralSeat	−0.78	0.45	[−2.07, −0.03]	0.04
ERI_status	−1.57	0.45	[−2.85, −0.81]	< 0.001
Gender	0.40	0.45	[−0.37, 1.68]	0.33
TypeElectoralSeat × ERI_status	2.76	0.90	[1.24, 5.33]	< 0.001
TypeElectoralSeat × Gender	0.14	0.90	[−2.43, 1.68]	0.87
ERI_status × Gender	−0.46	0.90	[−3.02, 1.07]	0.59
TypeElectSeat × ERI_status × Gender	3.43	1.79	[0.38, 8.57]	0.026

*Note:* The following coding was used: type of electoral seat (−0.5 = hard‐to‐win; 0.5 = easy‐to‐win), participants' ERI status (−0.5 = minority; 0.5 = majority), participants' gender (−0.5 = woman; 0.5 = man).

Moreover, in order to evacuate the possibility of a confounding effect of the political orientation measure when assessing the effect related to ERI status, we tested the above model while controlling for participants' political orientation. Indeed, as different survey showed, and as we also found in our own data (*p* < 0.001), minority participants are more politically left‐oriented compared to majority participants. Taking into account participants' political orientation did not change the effect of the ERI status (*p* < 0.001 for the ERI status and type of seat interaction; and *p* = 0.032 for the three‐way interaction), implying that the ERI status plays a role beyond political orientation. No moderation effects by participants' gender were found for change potential or candidate traits.

### 4.4 Discussion

The main findings supported H1. We found that minority participants were more likely to choose the minority candidate to run in a hard‐to‐win district rather than in one that was easier to win. Moreover, minority participants were more inclined to select the minority candidate for the hard‐to‐win district than the majority participants were. Attempting to explain the sociopsychological underpinnings of these findings, we only found partial support for H2. ERI minority participants, more than the majority ones, saw the minority candidate as better able to bring about change, and evaluated him more positively (as more competent and more communal). In part because of these views, they were overall more likely compared to majority participants to choose the minority candidate, independent of whether the election seat was hard or easy‐to‐win. However, to strengthen the interpretation of this (partial) mediation process and to address the limitations that the method of measurement‐of‐mediation poses (e.g., potential confounded variables), future studies could manipulate the mediator or adopt implicit mediation designs (Bullock and Green 2021).

Finally, it is worth noting that exploratory analyses showed an interaction with participants' gender revealing interesting nuances of the choice pattern. Indeed, among men, a glass cliff was found specifically among ERI minority individuals. ERI minority men were more likely to choose the minority candidate in the hard‐to‐win versus easy‐to‐win electoral district. In contrast, majority men showed the reverse pattern, being more likely to choose the minority candidate in the easy‐to‐win versus hard‐to‐win district. As for women, they were more likely to choose the minority candidate in the hard‐to‐win (vs. easy‐to‐win) electoral district, regardless of their ERI status. This means that both ERI minority and majority women showed a glass cliff.

## 5 Study 2

Study 2 aimed to replicate the primary findings, specifically that minority participants exhibit a glass cliff–type choice pattern, favouring the minority candidate over the majority candidate in a hard‐to‐win electoral district. We sought to enhance the relevance of this replication in three ways. First, we targeted a broader population, moving beyond the student cohort we used in Study 1. Second, we further explored participants' self‐identification as minorities, going beyond the criteria used Study 1. Finally, Study 2 was preregistered: https://aspredicted.org/blind.php?x=ASG_SQY.

### 5.1 Method

#### 5.1.1 Participants

Based on the results obtained in Study 1, we aimed to recruit a sample size allowing us to have 80% power to capture a minimum effect size of an OR = 6 (with a probability under H0, *p*1 = 0.7) for the effect of type of electoral seat among the minority participants. A G*Power 3 (Faul et al. 2007) analysis indicated that we needed approximately 92 minority participants. To increase the chances of having this number in our final sample, we decided to recruit a total sample of 400 participants. Moreover, this sample should be sufficient in terms of statistical power for the test of the mediated moderation model. Our reasoning followed the recommendation of Schoemann, Boulton, and Short (2017) in that we determined the sample size for each component of the indirect effect (as described in Muller et al. 2005). For the effect of participant ERI status on potential of change, we estimated a small effect size of *d* = 0.30, which requires 352 participants to have 80% power. For the interaction effect between potential for change and type of seat on the choice of the minority candidate, we estimated a small effect size of OR = 2 (with a probability under H0, *p*1 = 0.6), which requires 308 participants to have 80% power.

A total sample of 448 French participants were recruited in 2021 via the Crowd Panel platform (*M*_age_ = 39.12, SD_age_ = 12.60). We excluded 73 participants: those younger than 18 years old, those who did not give full consent and those who did not answer the comprehension check and attention check items (described below) correctly. The final sample consisted of 375 participants (197 women, 178 men; *M*_age_ = 39.16, SD_age_ = 12.68). Our sample was normally distributed on the political orientation scale (*M* = 4.77, SD = 1.87, 1 *left wing* to 9 *right wing*) and reported a moderate interest in French politics (*M* = 4.71, SD = 1.74, 7‐point scale), with 90.4% being registered on the French electoral lists.

In the assessment of participant ERI status, we first clarified our definition of belonging to an ERI minority in France as referring to ‘individuals who view themselves as a minority due to their skin colour, religion, ethnicity or their European or non‐European immigrant status (either as immigrants themselves or as descendants of immigrants)’ and then asked participants to rate the extent to which they considered themselves as belonging to an ERI minority on a scale from 1 (*not at all*) to 7 (*entirely*). As preregistered, we categorised participants into two distinct groups: those selecting 1 were classified as ‘majority participants’ (*N* = 243), while those selecting any number from 2 to 7 were classified as ‘minority participants’ (*N* = 132). This categorisation was informed by the highly skewed distribution of responses, with a large majority of participants concentrated at the lower end of the scale. Moreover, treating the variable as continuous would inaccurately suggest that the difference between choosing 1 (indicating ERI majority identification) and 2 (indicating partial ERI minority identification) is equivalent; for instance, to the difference between choosing 2 and 3 (both indicating some degree of ERI minority identification). We contend that individuals who chose any number from 2 to 7 share greater similarities in their minority self‐identification compared to those who chose 1, identifying as part of the majority. Thus, our approach conceptually opposes two groups: the majority (Level 1) and the minority (Levels 2–7).

#### 5.1.2 Procedure

We adopted the same procedure as in Study 1, randomly assigning participants to one of the two experimental conditions: ‘easy‐to‐win’ (*N* = 189) or ‘hard‐to‐win’ (*N* = 186). We included an attention check where participants were asked to tick the middle choice (4) on a 7‐point Likert scale. Then candidates were evaluated on candidate traits and signalling potential. Finally, participants were asked to nominate one of them to represent Party X for an electoral seat in District A. At the end, they answered various sociodemographic questions (such as gender, age, nationality, interest in French politics, political orientation and electoral registration status), were fully debriefed and provided their informed consent for us to use their data.

#### 5.1.3 Measures

Participants responded to each measure on a 7‐point scale. Unlike Study 1, where participants evaluated each candidate on individual agreement Likert scales, Study 2 used a direct comparative approach by applying bipolar scales. Thus, participants compared the two candidates across various characteristics with the candidate names indicated at the opposing ends of the scale (left–right orientation for the two candidates Olivier and Abdel was randomised across measures). The midpoint of the scale (4) indicated ‘both equally’. To mitigate potential reluctance, we explained in the instructions that when choosing between two people, it was common to think, ‘this one excels in this aspect’ or ‘the other is better in that aspect.’ For consistency across measures, we re‐coded measures such that a score greater than 4 indicates an evaluation favouring Abdel, and a score less than 4 indicates an evaluation favouring Olivier.

Candidate Traits. As in Study 1, we evaluated participants' perceptions of the candidates on competence, sociability and morality. In addition, we introduced two new dimensions: agency and effort. We conducted an exploratory factor analysis (extraction method: principal axis factoring, with a direct oblimin rotation), which revealed three factors: communion, regrouping sociability and morality (i.e., likeable, friendly, warm, honest, sincere and trustworthy; *α* = 0.87; *M* = 3.97, SD = 0.61); instrumentality, regrouping competence and agency (i.e., competent, intelligent, skilled, ambitious, competitive and self‐confident; *α* = 0.70; *M* = 3.98, SD = 0.57); and effort (i.e., persistent, tenacious and combative; *α* = 0.78; *M* = 4.54, SD = 0.91).

Change Potential Associated With the Candidates. Based on the exploratory factor analysis from Study 1, we used five items to assess the candidates' potential for change, including perceptions that [the candidate] ‘will signal to voters that Party X wants things to change’, ‘will represent the beginning of a new era for Party X in the constituency’, ‘will show the public and other parties that Party X has adopted changes in its operations’, ‘will conduct his political campaign in this district differently than his predecessors’ and ‘is likely to bring new and different ideas compared to his predecessors’. An exploratory factor analysis (using principal axis factoring and direct oblimin rotation) conducted on the current sample supported the one‐factor solution found in Study 1 (*α* = 0.85, *M* = 4.62, SD = 1.03).

Choice of the Candidate. The participants were asked to choose either the minority candidate or the majority candidate as a party nominee for electoral District A. Two participants did not indicate any choice.

### 5.2 Results for Main Hypotheses Tests

#### 5.2.1 Choice of the Candidate

We conducted a binary logistic regression with choice of candidate as the dependent variable (0 = Olivier, 1 = Abdel), and type of electoral seat (hard‐to‐win = −0.5, easy‐to‐win = 0.5), participant ERI status (minority = −0.5, majority = 0.5) and the interaction term as the predictors. The hypothesised two‐way interaction between type of electoral seat and participant ERI status was not significant, *B* = 0.38, 95% CI [−0.47, 1.24], *χ* ^2^(1, *n* = 373) = 0.774, *p* = 0.379, OR = 1.47, nor were the main effects (*p*s > 0.358).

#### 5.2.2 Candidate Evaluation

For the subsequent dependent variables, we conducted linear regression analyses, using the same predictors. To render the test for the intercept more relevant, we subtracted 4 from each score of the dependent variables, meaning that now the response option ‘both equally’ was assigned 0. Thus, the test for the intercept intuitively indicates whether the evaluation favours Abdel (i.e., positive values) or Olivier (i.e., negative values). This transformation did not impact the tests for the other effects.

Change Potential. Regressing the change potential score on the predictors only revealed that overall, regardless of ERI minority status and type of seat, participants associated a higher change potential with Abdel compared to Olivier, *B* = 0.62, 95% CI [0.51, 0.73], *t*(371) = 11.10, *p* < 0.001, *η*_*p*_^2^ = 0.25. No other effect reached significance (all *p*s > 0.521).

Candidate Traits. On the effort dimension, we found that overall, regardless of ERI minority status, and type of seat, participants perceived Abdel as being more tenacious and more combative than Olivier, *B* = 0.54, 95% CI [0.44, 0.64], *t*(371) = 10.91, *p* < 0.001, *η*_*p*_^2^ = 0.24. No effects were found for communion (all *p*s > 0.370) or instrumentality (all *p*s > 0.449).^5^

### 5.3 Exploratory Analyses: Participant's Gender and Political Orientation

**FIGURE 6:**
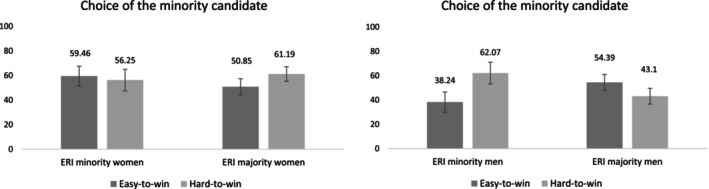
Estimated percentages with standard errors for the choice of the ERI minority candidate by type of seat and participant ERI status among women and men in Study 2.

As in Study 1, in exploratory non‐preregistered analyses, we also included participants' gender (women = −0.5; men = 0.5) and the associated interactions in the model (the results are presented in Table 2). For the dependent variable choice of the candidate, results showed a three‐way interaction effect between gender, type of seat and ERI status, *B* = 1.98, 95% CI [0.25, 3.72], *χ* ^2^(1, *n* = 373) = 5.01, *p* = 0.025, OR = 7.23. No other effects reached significance (all *p*s > 0.170). We decomposed the interaction by participant gender (Figure 6). For women, the effects of type of seat, ERI status and their interaction were all nonsignificant (all *p*s > 0.363). For men, the two‐way interaction between ERI status and type of seat was significant, *B* = 1.43, 95% CI [0.18, 2.70], *χ* ^2^(1, *n* = 373) = 4.43, *p* = 0.026, OR = 4.16. Although the simple effects did not reach significance (*p*s > 0.062), the pattern of percentages mirrors the results from Study 1. Specifically, minority men, but not majority men, were more likely to choose the minority candidate in the hard‐to‐win district than in the easy‐to‐win district. Furthermore, as in Study 1, we controlled for participants' political orientation. The effects related to participants' ERI status did not change (*p* = 0.034, for the three‐way interaction). No moderation effects by participants' gender were found for change potential or candidate traits.

**TABLE 2:** Logistic regression results for type of electoral seat, participants' ERI status, and gender on candidate choice in Study 2.

	*B*	SE (*B*)	95% CI	*p*
TypeElectoralSeat	−0.20	0.22	[−0.64, 0.23]	0.36
ERI_status	−0.06	0.22	[−0.50, 0.37]	0.77
Gender	−0.30	0.22	[−0.74, 0.13]	0.17
TypeElectoralSeat × ERI_status	0.44	0.44	[−0.43, 1.31]	0.32
TypeElectoralSeat × Gender	−0.11	0.44	[−0.99, 0.75]	0.80
ERI_status × Gender	0.02	0.44	[−0.85, 0.88]	0.97
TypeElectSeat × ERI_status × Gender	1.98	0.88	[0.25, 3.72]	0.025

*Note:* The following coding was used: type of electoral seat (−0.5 = hard‐to‐win; 0.5 = easy‐to‐win), participants' ERI status (−0.5 = minority; 0.5 = majority) and participants' gender (−0.5 = woman; 0.5 = man).

### 5.4 Discussion

The results did not support H1, and the expected interaction between the type of electoral seat and participant ERI status was not significant. Regarding candidate evaluations, the ERI minority candidate received a more favourable overall assessment in terms of change potential and effort. In essence, he was perceived as having a greater capacity to instigate change within the party and as being more combative, persistent and tenacious. However, such favourable evaluations were observed among both minority and majority participants, and regardless of the type of seat. Therefore, the prerequisites for the predicted mediation were not met and H2 could not be supported. This lack of effect on the candidates' evaluations might be due to the way we measured them, using a bipolar scale comparing the two candidates, rather than independent scales as in Study 1. Such measures may lead participants to tend towards the midpoint (i.e., ‘both equally’), thereby avoiding making a choice. For instance, on the candidate's traits items, around 50% of the participants chose the midpoint option.

Finally, it is worth noting that, similarly to Study 1, exploratory analyses showed a three‐way interaction between the type of electoral seat, participant ERI status and participants' gender. Specifically, there was a stronger preference for the ERI minority candidate in the hard‐to‐win district compared to the easy‐to‐win district among male ERI minority participants, but not among male ERI majority participants. No effects emerged among women, who overall presented a slight preference for the ERI minority candidate.

## 6 General Discussion

This research aimed to uncover one of the factors influencing the nomination of ERI minority candidates in politics and examined how decision makers' ERI status played a role in this process. We hypothesised the emergence of a glass cliff pattern, (i.e., the choice for the ERI minority candidate versus an ERI majority candidate for a hard‐to‐win seat) specifically among the ERI minority participants (H1). We reasoned that this would happen *because* ERI minority participants are likely to perceive a greater electoral potential in the ERI minority candidate compared to majority participants (H2). We tested these hypotheses in two experimental studies in which participants, playing the role of political party leaders, made decisions regarding candidate nominations either in easy‐to‐win or in hard‐to‐win electoral districts.

Study 1 provided support for H1, indicating that the glass cliff phenomenon specifically emerged among ERI minority participants. When seeking to explain these findings, we found partial support for H2. ERI minority participants were more likely than the majority participants to view the minority candidate positively and as a catalyst for change, leading to a higher probability of choosing him. However, this favourable evaluation was consistent regardless of the winnability of the electoral seat, limiting the explanatory scope of this potential mechanism for the higher probability of choosing the minority candidate in hard‐to‐win districts specifically.

Study 2 did not replicate the results from Study 1 concerning H1 nor did it support H2. Nevertheless, consistent with Study 1, the results showed a positive evaluation of the ERI minority candidate across all participants, regardless of their ERI status or the type of electoral seat. At this point, one may question the differences between the participant samples that could potentially explain why we did not replicate H1 in Study 2. The sample in Study 1 is a student sample, which may differ in various ways from the more general population sample in Study 2. However, the student sample is composed of students enrolled in IUT. The IUTs in France are public institutions within universities that offer 2‐year vocational degrees. Because they offer an accessible and practical route to employment, IUTs are known for attracting a diverse range of students from various socioeconomic and ethnic backgrounds. This diversity is evident in our sample, with 129 participants categorised as ERI minorities and 132 as ERI majority. Moreover, as regards political orientation—an ideological variable relevant to our studies—the samples exhibit relatively similar distributions, with very close means and standard deviations. Without engaging in extensive speculation, the major difference between the studies relates to the procedure. In Study 1, we used a paper‐pencil questionnaire, which was administered by a collaborator of the research team at the end of a regular lecture. In Study 2, the participants were recruited via the platform Crowd Panel. Thus, the conditions of administration were more closely supervised in Study 1.

Nevertheless, it is important to note that when we include participants' gender, the patterns of results are quite similar between the studies. Indeed, although not hypothesised a priori, exploratory analyses revealed the expected pattern in an interaction with participants' gender. In particular, a consistent pattern emerged across both studies concerning men. Minority men preferred the minority candidate for the hard‐to‐win electoral district, whereas majority men were more likely to choose the minority candidate for the easy‐to‐win district. Women (both ERI minority and majority) either preferred the minority candidate in the hard‐to‐win district (vs. the easy‐to‐win one, Study 1) or did not show any preference (Study 2). Although speculative at this point, and requiring further testing, one possible explanation aligns with our reasoning in H2, namely that minority men and both minority and majority women may perceive the minority candidate as possessing the qualities needed to succeed in a hard‐to‐win district, whereas majority men do not; hence, opting for the easier‐to‐win district. An alternative explanation is based on the outgroup homogeneity effect (Mullen and Hu 1989). Participants may be more likely to judge candidates from the opposite gender primarily by their social category, while giving more attention to the personal characteristics of candidates from their gender ingroup. Therefore, men might be more inclined than women to place male candidates in the political contexts they believe are more appropriate. However, these explanations should be tested in future studies, which we believe should place more emphasis on intersectionality to extend our understanding of political choice (e.g., Silva and Skulley 2019), particularly in the context of a political glass cliff.

A boundary condition for our result to appear may be the present experimental manipulation, in which the margins by which participants' parties won or lost were quite small (around 5%), thus inferring a ‘battlefield’ condition (see Thomas and Bodet 2013) where the possibility of winning becomes likely and thus may have elicited benign motives. It would be interesting in future research to also test a ‘stronghold’ condition in which the margin is much larger and the likelihood of losing greater, potentially impacting the decision makers' motives to choose an ERI minority candidate (see experimental variations in Aelenei et al. 2020).

### 6.1 Theoretical Implications and New Avenues of Research

To understand the general underrepresentation of ERI minorities in politics, we argue for shifting the focus from investigating voter biases (Auer, Portmann, and Tichelbaecker 2023) to examining how parties nominate candidates. As already demonstrated (Kulich, Ryan, and Haslam 2014; Robinson et al. 2024), ERI minority candidates may struggle to get elected because they tend to be assigned to precarious glass cliff positions, where their likelihood of winning the seat is reduced (Kulich, Ryan, and Haslam 2014). The present research aimed at extending our understanding of the social psychological underpinning that could account, at least in part, for this phenomenon. Past experimental research demonstrated that left‐wing participants tended to produce the glass cliff for ERI minorities (Aelenei et al. 2020), suggesting that decision makers with more positive attitudes towards ERI minorities may, in certain contexts, be more likely to assign minorities to hard‐to‐win electoral districts. Based on the exploratory findings in the present studies, we observe similar patterns among ERI minority men, beyond the effect of political orientation. One possible explanation for this effect relies on intra‐minority solidarity and the common ingroup identity model (Burson and Godfrey 2020; Gaertner et al. 1993). Thus, viewing a candidate with ERI minority origins as an ingroup member of a minority superordinate group, which includes individuals of any ERI minority background, may produce a more favourable rating of this candidate among ERI minority decision makers. Their decision to assign ERI minorities to difficult electoral tasks is thus likely motivated by the belief that these minorities are the most capable people to reverse the unfavourable electoral trend. This explanation is supported by research indicating that female recruiters, but not male recruiters, are especially likely to appoint a female candidate (i.e., an ingroup member) in a high‐risk position, as opposed to a low‐risk position (Hunt‐Earle 2012). In summary, political glass cliff scenarios involving ERI minorities can be influenced by left‐leaning and ERI minority decision makers alike, although the underlying motives are likely to differ. On the one hand, left‐wing individuals may perceive ERI minorities as particularly capable of reversing unfavourable odds, given their generally egalitarian worldviews and endorsement of humanitarian values (Caprara et al. 2006). On the other hand, ERI minorities are likely to be motivated by inter‐minority solidarity and a sense of a common ingroup identity when prioritising ERI minority candidates to improve the situation.

At first sight, these results point to a paradox. In attributing political qualities to ERI minorities, decision makers may inadvertently lead them into a pitfall. However, research has also shown that hard‐to‐win seats are not always related to more failure. For instance, an analysis of the 2011–2016 legislative elections in the United States showed that women in both the Republican and the Democratic parties faced a glass cliff in House races, as they were more likely to run for hard‐to‐win seats (Robinson et al. 2021). Of importance, this only resulted in Republican women being more likely than their male counterparts to fail. Despite female Democrats running for a higher proportion of hard‐to‐win seats than male Democrats, they still managed to win as many seats as their male colleagues. Hard‐to‐win seats are therefore not necessarily more precarious per se. Other factors may influence winnability. For instance, the support and the resources a candidate may obtain from their party may mitigate the potentially unfavourable electoral situation (Robinson et al. 2021; Sanbonmatsu 2018). When party officials believe that someone will be capable of reversing unfavourable electoral odds, they will ensure they are in the best condition to succeed.

## 7 Conclusions

Political glass cliffs are the result of a variety of mechanisms. Experimental studies such as those in the present work help disentangle social psychological processes in individual decision‐making processes. However, in actual political elections, many other factors associated with the political system, party landscape, positive discrimination policies or diversity norms may interact with, or override, individuals' personal preferences. Each research methodology, using either archival, experimental, or qualitative data, allows us to understand certain facets of the glass cliff while remaining ignorant of others. The present research added theorising and partial empirical support to one potential explanation, as concerns the role of decision makers' ERI minority background and more benign motivations that lead to the preference for ERI minority candidates in hard‐to‐win seats.

## Conflicts of Interest

The authors declare no conflicts of interest.

## Supporting information


**Data S1** Supporting Information

## Data Availability

The data that support the findings of this study are openly available in OSF at https://osf.io/s5yuv/?view_only=9740612250964b869af4adecb6c6117f.

## Endnotes

^1^
In Tolley (2019), individuals who identify as South Asian, Chinese, Black, Filipino, Latin American, Arab, Southeast Asian (e.g., Cambodian and Thai), West Asian (e.g., Iranian and Afghan), Korean, and Japanese were categorised as racialised. In Van Trappen, Vandeleene, and Wauters (2021), immigrant origins referred to non‐European, Southern‐European or Eastern‐European origin.

^2^
The Supporting Information, the data, the codebook and the R scripts for the studies are available at https://osf.io/s5yuv/?view_only=9740612250964b869af4adecb6c6117f.

^3^
Twelve participants did not indicate any choice, and four participants chose both candidates.

^4^
Since the minority candidate was described as being of Maghreb origin, we wanted to verify that the observed effects were not solely due to the presence of participants of Maghreb origin in our sample (38 out of 313), possibly because of specific in‐group identification. Excluding these participants did not change the hypothesised interaction effect, *p* = 0.001, thus supporting our explanation of intra‐minority solidarity.

^5^
In the evaluation of candidates' traits, a significant proportion of participants (i.e., around 50%) opted for the midpoint on the scale, leading to nonnormal distributions in the model residuals. To address this limitation, we employed bootstrapping techniques to validate the robustness of our findings. Given that the outcomes remained consistent, we report the conventional regression analyses.
