# Supplementary material for: The Political Glass Cliff: When Ethnic, Racial and Immigration Minority Participants Choose Minority Candidates for Hard‐To‐Win Seats
Source: J Community Appl Soc Psychol. 2024 Nov 20;34(6):e70014. doi: 10.1002/casp.70014 (PMC11586809; doi:10.1002/casp.70014)
Supplement: Supplementary file 1 — Data S1 Supporting Information [file CASP-34-e70014-s001.docx]

## Supplementary Materials

## SM1. Community and Social Impact Statement

Ethnic minority groups remain underrepresented in politics in many European countries. In the present research, we investigated a factor that may contribute to this underrepresentation by examining who chooses members of these groups to run for political office and under what conditions. We found evidence of a political "glass cliff," where ethnic minority candidates are more likely to be nominated for elections in hard-to-win seats. Importantly, we showed this effect in particular for male voters with ethnic minority backgrounds. This finding suggests that the glass cliff may not, as sometimes argued, be motivated by a desire to see the atypical candidate fail. Instead, in the circumstances described, it may reflect decision-makers' belief in the candidate’s potential. Indeed, our research shows that participants view minority candidates as having greater potential for change, higher competence, and stronger communal qualities.


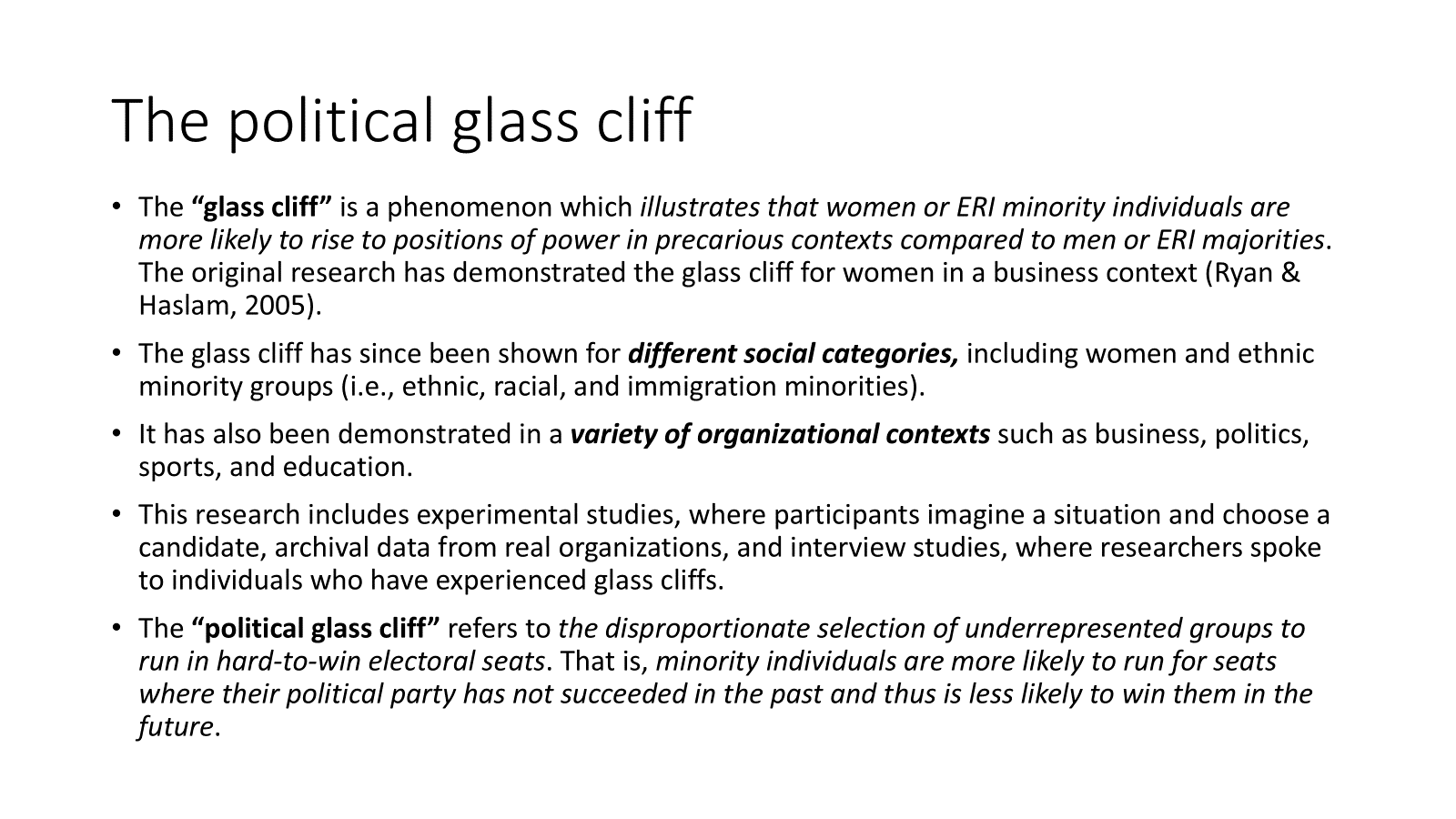


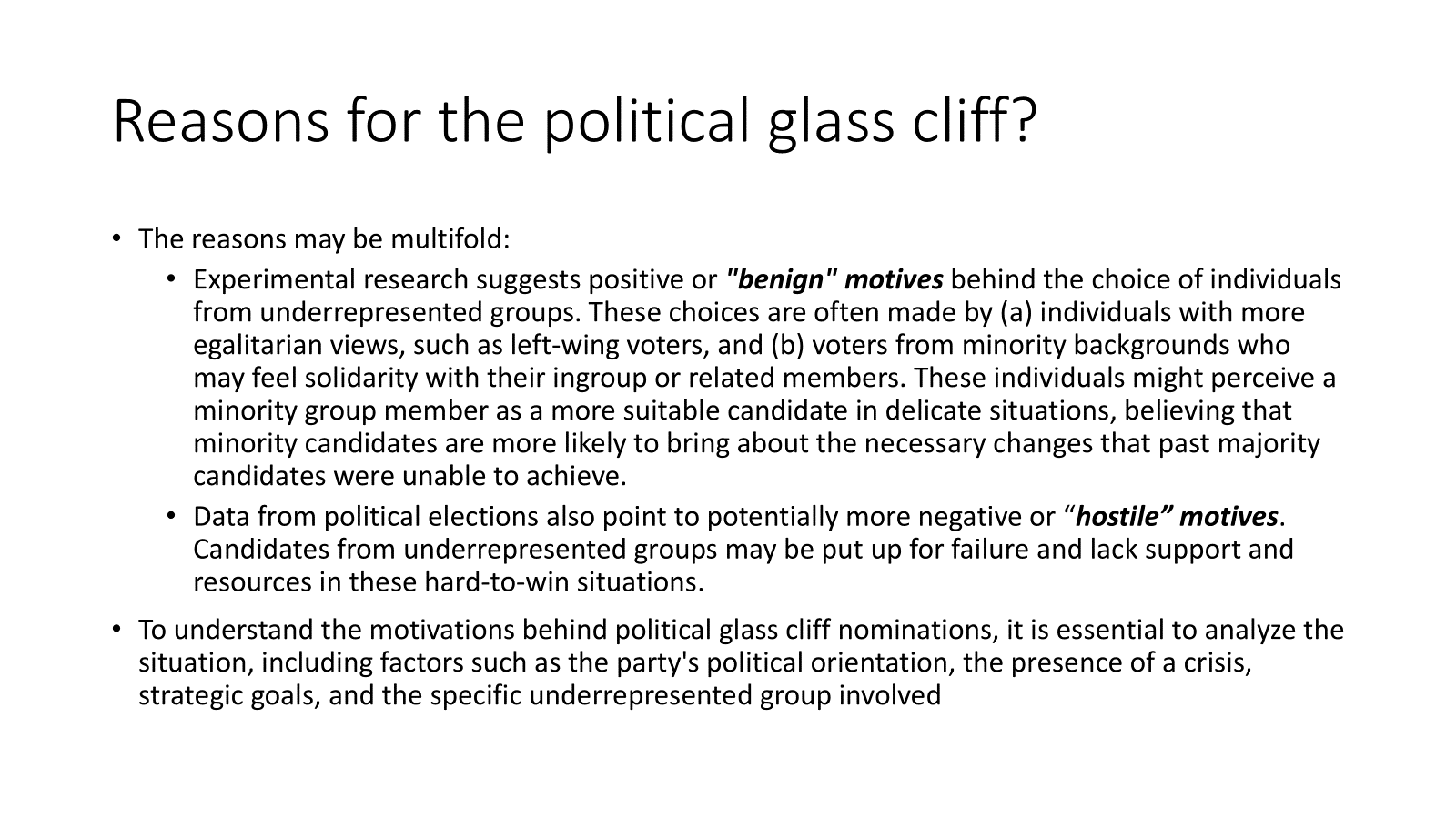


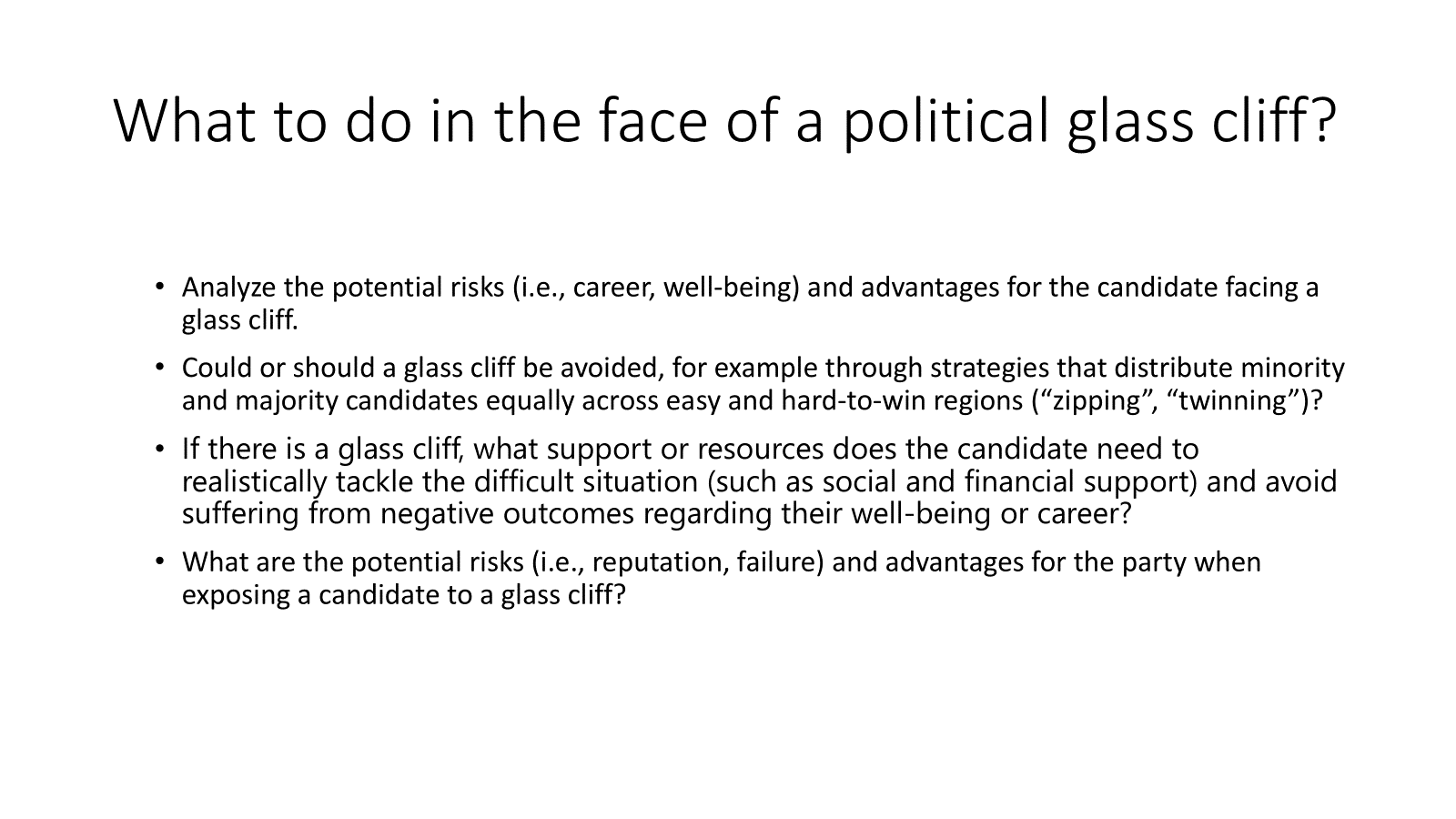


## SM2. Pilot Study

### Method

#### Participants

A total sample of 203 students from a university in Paris participated in the study. We excluded participants younger than 18 years old, who did not consent to their responses being used for research purposes, and who did not correctly answer the comprehension checks (detailed below). Based on these exclusion criteria, the final sample consisted of 180 participants (154 women, 26 men; *M_age_* = 20.63, *SD_age_* = 1.45). Forty-one of them considered themselves an ethnic minority in France and 139 did not (see below). In terms of political orientation, on a scale from 1 *left-wing* to 9 *right-wing*, our sample presented a normal distribution (*M* = 4.09, *SD* = 1.67). Participants also reported on a 7-point Likert scale their interest in French politics (*M* = 4.81, *SD* = 1.74).

#### Procedure

The study was conducted in person by the experimenter at the end of a regular class. We followed the scenario-based procedure described in Aelenei et al. (2020). Participants first provided their political orientation and indicated the French political party they felt closer to. They were then given the general instruction to take on the role of Party X’s leader. As the party leader, they were required to nominate a candidate in a specific electoral district (i.e., District A) for the following legislative elections. Depending on the experimental condition, the electoral district was presented as either an “easy-to-win" or “hard-to-win" seat for Party X. In the “easy-to-win" condition (*n* = 88), a graph showed that Party X had won the previous election in the district with 54.6% of the vote, compared to Party Y’s 46.4%. Conversely, in the “hard-to-win" condition (*n* = 92), the graph illustrated Party X losing the last election with 46.4% of the vote, while Party Y secured 54.6%. Two check items were presented after the experimental manipulation. The first verified whether participants accurately read the graph and understood which party (X or Y) had won the last election in the district. Those who answered incorrectly were excluded. The second item evaluated participants’ perceptions of Party X's chances to win the upcoming election in the district. It queried which party was deemed most likely to win (X or Y, both, or neither). In the “easy-to-win" condition—where Party X had won previously—responses indicating Party Y or neither party as most likely to win led to exclusion. Similarly, in the “hard-to-win" condition—where Party X had lost—participants who believed Party X had the highest chances were also excluded.

Next, participants were informed that as Party X’s leader, they would evaluate two candidates and nominate one to represent the party for an electoral seat in District A. To heighten engagement with the task, participants were told, “*We understand that making judgments based on limited information is challenging, but it is your initial impression that we are interested in*.” The candidate profiles (based on Aelenei et al., 2020) were comparable in education (master’s degree in political science), professional background (management level position), and hobbies. The sole distinguishing feature was the candidate’s ERI minority status. The ERI minority candidate, Abdel Benzekri, had a typically North African name, was born in Algeria, and had lived in France since age five. The ERI majority candidate, Olivier Chapuis, had a typically French name, and had lived in France all his life. The descriptions of the two candidates were presented in a counterbalanced order. After the presentation of each candidate, participants were asked to choose the candidate they wished to nominate for District A. They then rated the potential for change and the perceived competence of the chosen candidate. Finally, participants completed several sociodemographic items, including their ethnic background. Specifically, they reported whether they considered themselves as an ethnic minority in France (Yes/No). Other demographics included gender, age, nationality, interest in French politics, and voting status. They also indicated the inferred political orientation of Party X and which existing French party they believed was closest to Party X. They reaffirmed their consent for their responses to be used for research purposes, were debriefed, and thanked for their participation.

#### Measures

Choice of the Candidate. The participants were asked to indicate whom they chose as their party's nominee to run for election in District A (the minority candidate versus the majority candidate). One participant did not indicate a choice.

**Change Potential.** To assess the change potential associated with the chosen candidate, we included seven items (adapted from Aelenei et al., 2020, measured on a 7-point Likert scale) that captured both the actual the candidate could implement (e.g., “the candidate will conduct his political campaign in this district differently than his predecessors”*)* and the symbolic changes *that the candidate choice could* signal *(*“the candidate will signal to voters that Party X wants things to change”). An exploratory factor analysis (extraction method: principal axis factoring, with a direct oblimin rotation) conducted on the seven items revealed a two-factor solution with eigenvalues greater than 1. All items, with the exception of item 7, loaded on the first factor (all loadings greater than .52). Item 7, which was the only item phrased in reverse (i.e., “The policy of this candidate will be similar to that of the candidates from Party X who ran in this constituency in the previous elections”), loaded on the second factor (.85). Consequently, we used the six items loading on the first factor to create a single scale measuring general change potential and excluded Item 7 (*M* *= 5.00*, *SD* *= 1.11,* α *= .84)*.

**Perceived Competence.** We measured the perceived competence of the chosen candidate with the following single item: “Because of his experience, the candidate is able to persuade the voters” (*M* = 5.44, *SD* = 1.36). The correlation between the change potential and the perceived competence of the chosen candidate was positive and significant, *r*(176) = .26, *p* = .001.

### Results

#### **Candidate Choice**

To test for H1, we conducted a binary logistic regression with choice of the candidate as the dependent variable (1= choice of the minority candidate; 0 = choice of the majority candidate). We included as predictors: type of electoral seat (-0.5 = hard-to-win; 0.5 = easy-to-win), participant ERI status (-0.5 = minority; 0.5 = majority) and their interaction. Results showed that neither the main effect of type of seat, *B* = 0.30, *SE* = 0.40, *χ*^2^(1, *n* = 180) = 0.59, *p* =.444, *OR* = 1.35, 95% CI [0.62, 2.95], nor the main effect of ERI status, *B* = 0.09, *SE* = 0.40, *χ*^2^(1, *n* = 180) = 0.59, *p* =.818, *OR* = 1.10, 95% CI [0.50, 2.38], were significant. Moreover, the expected Type of seat × Participant ERI Status interaction was not significant, *B* = 1.08, *SE* = 0.79, *χ*^2^(1, *n* = 180) = 1.85, *p* =.174, *OR* = 2.94, 95% CI [0.62, 13.89]. However, a closer look at the likelihoods of choice indicated that the pattern goes in the direction predicted in H1. Among minority participants, the likelihood of choosing the minority candidate was higher in the hard-to-win electoral district (83.20%) than in the easy-to-win electoral district (47.90%). Among majority participants, the likelihood of choosing the minority candidate was higher in the easy-to-win electoral district (71.14%) than in the hard-to-win electoral district (60.59%).

#### Candidates Evaluations

**Change Potential****.** We ran an ANOVA on change potential associated with the chosen candidate, with type of electoral seat, participant ERI status, chosen candidate, and their interactions as predictors. The analysis revealed a main effect of the type of electoral seat, where change potential was higher in the easy-to-win electoral district (*M* = 5.00, *SE* = 0.12) than in the hard-to-win electoral district (*M* = 5.63, *SE* = 0.15), *F*(1,168) = 4.02, *p* = .047, η_p_^2^ = .02. The main effect of chosen candidate was also significant, *F*(1,168) = 59.95, *p* < .001, η_p_^2^ = .26. When the chosen candidate was the minority one, change potential was higher than when the chosen candidate was the majority one (*M* = 5.54, *SE* = 0.10, and *M* = 4.09, *SE* = 0.16, respectively). The interaction effect between the Chosen candidate × Participant ERI status was not significant, *F*(1,168) = 3.73, *p* = .055, η_p_^2^ = .02. However, looking at the pattern of means showed that minority participants associated the chosen minority candidate (*M* = 5.78, *SE* = 0.18) with more change potential than majority participants (*M* = 5.29, *SE* = 0.10), *F*(1,168) = 5.62, *p* = .019, η_p_^2^ = .03. The change potential of the chosen majority candidate did not vary as a function of participant ERI status (*M_Minority_* = 3.98, *SE_Minority_* = 0.28, and *M_Majority_* = 4.21, *SE_Majority_* = 0.14), *F*(1,168) = 0.56, *p* = .457, η_p_^2^ < .01.

**Perceived Competence.** The same ANOVA was performed on the perceived competence of the chosen candidate. Results revealed a main effect of chosen candidate, *F*(1,168) = 4.87, *p* < .029, η_p_^2^ = .03. When the chosen candidate was the minority one, perceived competence was higher than when the chosen candidate was the majority one (*M* = 5.65, *SE* = 0.15, and *M* = 5.06, *SE* = 0.22, respectively). This effect was qualified by a significant Chosen candidate × Participant ERI status interaction, *F*(1,168) = 4.79, *p* = .030, η_p_^2^ = .03. Specifically, minority participants perceived the chosen minority candidate (*M* = 5.85, *SE* = 0.26) to be more competent than the chosen majority candidate (*M* = 4.68, *SE* = 0.40), *F*(1,168) = 6.13, *p* = .014, η_p_^2^ = .04. However, the majority participants did not perceive the chosen minority candidate (*M* = 5.44, *SE* = 0.14) to be more competent than the chosen majority candidate (*M* = 5.44, *SE* = 0.20), *F*(1,168) < 0.01, *p* = .986, η_p_^2^ < .01.

**SM3. Manipulation of the Type of Seat in Studies 1 & 2**

**[Common instructions]** Please imagine yourself in the following situation: The legislative elections are approaching and your political party, Party X, is preparing the nominations of candidates in the Île-de-France region. You are the leader of Party X in the Île-de-France region. You are therefore highly involved in party affairs and you want to attract a large number of voters. One of your tasks is to place the party's candidates in the various constituencies. In particular, you have to choose the candidate who will run in constituency A in Paris. Your final choice will be to select one of two candidates who will be presented to you later in the questionnaire. First of all, please take a look at the results of the last general election in constituency A in the second round.

**[Hard-to-win condition]**

**[Easy-to-win condition]**

**SM4. Exploratory Measures in Studies 1 & 2**

### Study 1

#### Potential to Persuade Voters

This measure (inspired by Aelenei et al., 2020) used a 7-point Likert scale to evaluate each candidate’s potential to persuade different categories of voters (i.e., voters in general, women, men, poor people, rich people, young people, ethnic minorities, old people). Two exploratory factor analyses (extraction method: principal axis factoring, with a direct oblimin rotation) revealed the same two-factor pattern for each candidate. A first factor referred to the candidates’ potential to persuade minority voters and regrouped the following categories of voters: ethnic minorities, poor people, and young people (*α*_Olivier_ = .68, *α*_Abdel_= .58; *M*_Olivier_ = 3.63, *SD*_Olivier_ = 1.03, *M*_Abdel_ = 5.41, *SD*_Abdel_ = 0.94). The second factor captured candidates’ potential to persuade majority voters and included: voters in general, women, men, rich people, old people (*α*_Olivier_ = .74, *α*_Abdel_= .79; *M*_Olivier_ = 5.13, *SD*_Olivier_ = 0.84, *M*_Abdel_ = 4.29, *SD*_Abdel_ = 0.97).

We implemented the same procedure as for the other dependent variables, by calculating difference scores: potential to persuade minority/majority voters associated with the minority candidate – potential to persuade minority/majority voters associated with the majority candidate. We regressed these difference scores on the predictors: type of seat, participant ERI status, participant gender, and all two-way and three-way interactions. Results showed that overall, the minority candidate (*M* = 5.41, *SE* = 0.06) was perceived as having greater potential than the majority candidate (*M* = 3.62, *SE* = 0.07) to persuade minority voters, *B* = 1.78, *SE* = 0.09, *t*(252) = 19.27, *p* < .001, 95% CI [1.60, 1.97], η_p_^2^ = .60. On the potential to persuade majority voters, results showed a three-way interaction between type of seat, participant ERI status and participant gender, *B* = 1.14, *SE* = 0.57, *t*(252) = 1.98, *p* = .048, 95% CI [0.01, 2.27], η_p_^2^ = .02. However, simple effects analyses revealed that the interaction effect between type of seat and participant ERI status was neither significant among male (*p* = .073) nor female participants (*p* = .367).

#### Right-wing Authoritarianism Scale

For exploratory purposes, we included a 5-item, 7-point Likert scale, measuring participants’ level of endorsement of right-wing authoritarianism (Duckitt et al., 2010). Since the internal consistency of the scale was very low (*α* = .47), and the exploratory factor analysis was inconclusive we did not conduct any further analysis on this measure.

### Study 2

To render the test for the intercept more relevant, we subtracted 4 from each score, meaning that now the response option “both equally” was assigned 0. Thus, the test for the intercept will inform us whether the evaluation favors Abdel (i.e., positive values) or Olivier (i.e., negative values). This transformation did not impact the tests for the other effects.

#### Potential to Persuade Voters

We used the same measure as in Study 1, assessing candidates’ potential to persuade voters in general, women, men, poor people, rich people, young people, ethnic minorities, and old people. An exploratory factor analysis revealed the same two-factor solution, namely candidates’ potential to persuade minority voters (*α* = .66; *M* = 5.18, *SD* = 0.90), and candidates’ potential to persuade non-minority voters (*α* = .73; *M* = 3.10, *SD* = 0.78). Participants perceived the minority candidate as having greater potential than the majority candidate to persuade minority voters, *B* = 1.19, *SE* = 0.05, *t*(367) = 24.33, *p* < .001, 95% CI [1.10, 1.29], η_p_^2^ = .62. Conversely, participants perceived the majority candidate as having greater potential than the minority candidate to persuade majority voters, *B* = -0.92, *SE* = 0.04, *t*(367) = -21.82, *p* < .001, 95% CI [-1, -0.84], η_p_^2^ = .57.

#### Candidate Atypicality

The perception of the atypicality of the candidates was measured with one item: “the candidate is an atypical candidate” (*M* = 5.13, *SD* = 1.31). Overall, participants perceived the minority candidate as representing a more atypical candidate than the majority candidate, *B* = 1.16, *SE* = 0.07, *t*(367) = 16.44, *p* < .001, 95% CI [1.02, 1.30], η_p_^2^ = .42.
